# Supplementary material for: Development of Injectable PEGylated Liposome Encapsulating Disulfiram for Colorectal Cancer Treatment
Source: Pharmaceutics. 2019 Nov 14;11(11):610. doi: 10.3390/pharmaceutics11110610 (PMC6920821; doi:10.3390/pharmaceutics11110610)
Supplement: Supplementary file 1 [file pharmaceutics-11-00610-s001.pdf]

pharmaceutics

# Supplementary Materials: Development of Injectable PEGylated Liposome Encapsulating Disulfiram for Colorectal Cancer Treatment

**Mohammad Najlah, Ammar Said Suliman, Ibrahim Tolaymat, Sathishkumar Kurusamy, Vinodh Kannappan, Abdelbary M.A Elhissi and Weiguang Wang**

## A.1 Release Studies

The release of DS from formulations was investigated using dialysis. Considering the loading efficiency, volumes of each liposomal formulation containing 0.5 mg of DS was toped up to 1 ml by purified water and placed in a dialysis tube (MWCO 3500) and tightly sealed. For free drug, 0.5 mg was dissolved in 1 ml of ethanol: water: tween 80 (50:49.9:0.1%). Then, the dialysis tube was immersed in 50 ml (total volume) release medium (PBS (pH 7.4) containing 0.1% (*v*/*v*) Tween 80) and incubated with stirring in for 24 h at 37 °C. Samples (0.2 ml) were taken at time intervals from the release medium for 24 h and replaced by a similar volume of fresh medium. The concentration of DS was determined by HPLC as above.

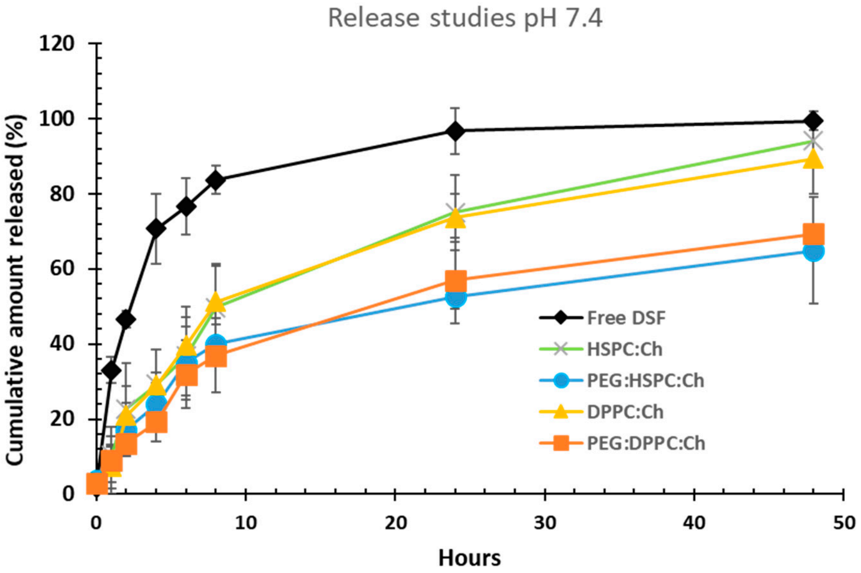


**Figure S1.** The release profiles of DS form liposomes.

## A.2 pH Measurements

The pH of liposomal formulations was determined using a Corning 220 pH meter (Cole-Palmer, Teddington, UK) previously calibrated using the provided pH 4 and pH 7 solutions.

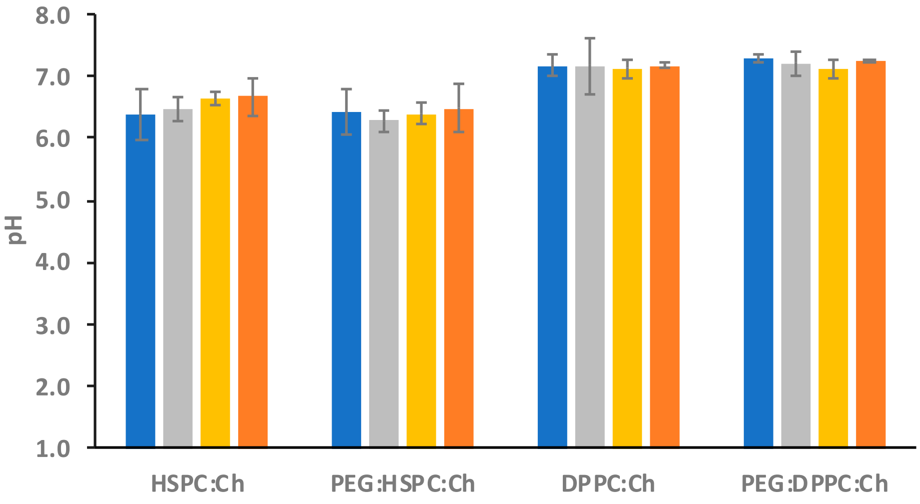


**Figure S2.** The pH values of DS liposomal formulations.

The effect of drug concentration, lipid composition and PEGylation on the pH of liposomes was also investigated. The pH of all HSPC liposomes was slightly acidic, while the pH of DPPC-liposomes was neutral (Figure 2); this difference in pH between both (HSPC liposomes and their correspondent DPPC formulations) was significant ($p < 0.05$). It is worth mentioning that the pH of formulations was not affected by DS concentration neither by PEGylation. For example, for HSPC liposomes, the pH of DS-free liposomes and that having the maximum DS concentration were 6.39 ± 0.4 and whereas the pH of liposomes was 6.67 ± 0.3 ($p > 0.05$). Similarly, the pH of DS-free PEG-HSPC liposomes was 6.43 ± 0.24 while the pH of liposomes with maximum drug concentration was 6.48 ± 0.39 ($p > 0.05$).

**A3 Survival curves of MTT cytotoxicity assay for the empty liposomal formulations**

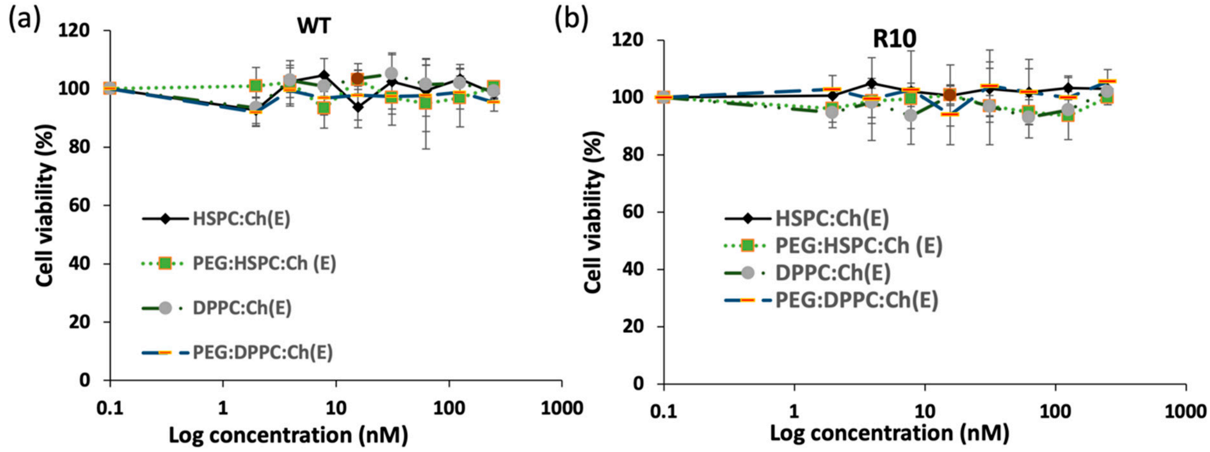


**Figure S3.** Survival curves of MTT cytotoxicity assay for the empty lipospmal formulations on colorectal cancer cell lines (**a**) H630WT and (**b**) H630R10 ($n = 3 \pm$ SD). "E" denotes "empty".
